# Synergy of Effectuation and Causation: An Emotional Complexity Perspective

**DOI:** 10.3389/fpsyg.2021.732936

**Published:** 2022-01-04

**Authors:** Huangen Chen, Qian Xu

**Affiliations:** School of Business Administration, Faculty of Business Administration, Southwestern University of Finance and Economics, Chengdu, China

**Keywords:** emotional complexity, cognitive flexibility, effectuation, causation, synergetic effect

## Abstract

This study enriches the literature on entrepreneurial decisions by investigating the antecedents of the synergetic use of causal and effectual logic. Based on entrepreneurial metacognition and emotional complexity theories, we argued that the emotional complexity of an entrepreneur, referred to as the granular experience of, or variety in, experienced emotions during the entrepreneurial task, would contribute to the synergetic use of decision logic. With survey data gathered from 218 Chinese entrepreneurs, we found that entrepreneurs with higher emotional complexity are more likely to adopt two types of entrepreneurial logic in tandem, and cognitive flexibility mediates this positive relationship. Thereby, this study helps to unravel some of the complexities behind the choice of decision logic of entrepreneurs.

## Introduction

Causation and effectuation are two disparate decision logics that entrepreneurs frequently use to cope with uncertainties (Sarasvathy, 2001; Alsos et al., 2020). Causation takes a particular effect as given and focuses on selecting between means^1^ to attain this effect, which is the characteristic of “many-to-one mappings,” in contrast, effectuation takes a set of means as given and focuses on selecting between possible effects of these means, characteristic of “one-to-many mappings” (Sarasvathy, 2001, 2008).

In her original works, Sarasvathy (2001) admitted that these two disparate decision logics are not mutually exclusive within an individual. Recently, investigators have observed that entrepreneurs often use causal and effectual combinations during their entrepreneurial process (Berends et al., 2014; Galkina and Lundgren-Henriksson, 2017; Galkina et al., 2021). They also found that the simultaneous or alternate engagement of two decision logics could contribute to their improved opportunity generation (Maine et al., 2015), business model innovation (Reymen et al., 2017), product innovation (Berends et al., 2014), and the venture performance (Smolka et al., 2018; Yu et al., 2018). Despite these benefits of synergy, we still know less about its antecedents, specifically, why some entrepreneurs could use the two decision logics in tandem and how they arrive at such a balance.

Our research employs entrepreneurial metacognition and emotional complexity theories to answer these questions. Entrepreneurial metacognition theory regards the entrepreneur as “a fully engaged thinker who has multiple cognitive strategies available and chooses among them based on goals, motives, and needs” (Fiske and Taylor, 1991, p. 13) to act (or not) in response to perceived opportunities (Haynie et al., 2010). Specifically, an entrepreneur uses the metacognition process, a kind of “higher cognition process,” to adjust their current-used cognitive strategy to accommodate changes in the environment or motivation (Flavell, 1979). Following this perspective, we regarded causal and effectual logic as two different forms of cognitive strategy^2^. Through the active engagement of the metacognitive process, an entrepreneur can be flexible in their choice of cognitive strategies (Haynie et al., 2012). Consequently, they would be more likely to recognize multiple alternatives to process a given task and consciously consider these alternatives (Mitchell et al., 2011).

Given the extreme context (in terms of time pressure and uncertainty) and high identification with the founded venture (Cardon et al., 2005), entrepreneurship is an emotional journey (Cardon et al., 2012). We further introduced emotional complexity as an antecedent to the synergetic use of causal and effectual logic. Emotional constructivism claims that emotion is not a response but an active prediction of internal and external events (Barrett, 2017b). The emotional experience of an individual is rarely pure and simple but often mixed and complex (Berrios, 2019). Emotional complexity is conceptualized as the ability to have a granular experience of, or variety in, experienced emotions during entrepreneurial tasks. Moreover, we argued that an entrepreneur that experiences a more complex emotional experience has a more accurate interpretation of the situation, more regulatory resources for metacognitive functioning, and a greater likelihood of reconciling two disparate cognitive strategies (causal and effectual) to achieve the desirable entrepreneurial outcome (Rees et al., 2013; Grossmann et al., 2019).

In summary, this research suggests that an entrepreneur with a complex emotional experience in his entrepreneurial task would be more flexible in evaluating alternative cognitive strategies and formulating one congruent with the changing environment.

A sample of 218 entrepreneurs from China supported our hypotheses. We found that entrepreneurial emotional complexity is positively related to causal and effectual logic synergy, and cognitive flexibility mediates this relationship.

We aspired to make three contributions through this study. First, the core contribution is introducing emotional complexity as a critical antecedent to the synergetic use of causation and effectuation, which addresses the shift in research focus from simple emotion to mixed/complex emotions (Gielnik et al., 2021). Moreover, we drew from the emotional complexity theory and confirmed the importance of mixed/complex emotion to the metacognitive processes of entrepreneurs, linking the previously unconnected core constructs by theorizing about the underlying theoretical mechanism (Shepherd and Patzelt, 2017). Finally, we advanced the emotional complexity literature by developing and empirically testing a novel theoretical link between emotional complexity and the selection of decision logic of an entrepreneur (Galkina et al., 2021).

## Theoretical Framework and Hypothesis

### Causation, Effectuation, and Their Synergy

Entrepreneurs can opt for different cognitive strategies (Kuratko et al., 2020) to cope with the uncertainties. Based on the attitudes toward means or effects, Sarasvathy (2001) conceptualized two types of heuristics used in entrepreneurial decision-making, namely, causation and effectuation.

Causation “takes a particular effect as given and focus on selecting between means to attain this effect” (Sarasvathy, 2001, p. 245), which is goal-driven and characteristic of “many-to-one mappings,” effectuation “takes a set of means as given and focus on selecting between possible effects that can be created with that set of means” (Sarasvathy, 2001, p. 245), which is non-goal or means driven and characteristic of “one-to-many mappings.” The former prefers to embark on the predictable aspects of an uncertain future, whereas the latter is controllable (Galkina et al., 2021).

Even though effectual logic describes a decision process distinct from causal logic, these disparate cognitive strategies are not mutually exclusive within an individual. Based on the conception of causation and effectuation suggested by Sarasvathy (2001), the set of “means” available to the entrepreneur is a common element of causal and effectual logic; the difference resides in the cognitive framework adopted to organize and act upon (Shepherd and Patzelt, 2017). The selection of causal or effectual reasoning partly depends on the extent to which the awareness of an entrepreneur and organization of these “means” (Haynie et al., 2010).

As “the condition of uncertainty is often not stable over time” (Alvarez and Barney, 2005, p. 789), the dynamics of the environment may influence the viability of goal-driven/causal logic or means-driven/effectual logic. Thus, the adoption of cognitive strategy of an entrepreneur is neither a single choice between effectuation and causation nor a linear trajectory of development from one to the other (Galkina et al., 2021). An entrepreneur, who recognizes two decision logics as alternative cognitive strategies and adaptably identifies the most appropriate one, is more likely to achieve his entrepreneurial goals.

Recently, empirical studies have confirmed that causation and effectuation could be adopted simultaneously or alternately under some conditions, and such synergy would be beneficial (Reymen et al., 2017; Smolka et al., 2018; Yu et al., 2018). Specifically, researchers found that the simultaneous or alternate engagement of two decision logics could contribute to their improved opportunity generation (Maine et al., 2015), business model innovation (Reymen et al., 2017), product innovation (Berends et al., 2014), and the venture performance (Smolka et al., 2018; Yu et al., 2018).

While combining causal logic and effectual logic is helpful for the success of a start-up, the road to their synergy is still vague. There are a few exceptions such as Reymen et al. (2015); Jiang and Tornikoski (2019), and Braun and Sieger (2021). Most of these are qualitative evidence. Based on a longitudinal case study of nine technology-based ventures, Reymen et al. (2015) found that changes in perceived uncertainty, resource position, and stakeholder pressure would adjust the scope of these ventures, which could lead to the shift and re-shifts of their decision logics. Through a comparative process study of four new technology-based ventures over 2 years at the founding team level, Jiang and Tornikoski (2019) found that through the cognitive interpretation of anticipated or unanticipated consequences encountered, an entrepreneur would perceive different uncertainties in their venture and then shift their decision logics. In addition, Braun and Sieger (2021) employed the lens of family obligation and confirmed that the family financial support of university entrepreneurs would be related to their ambidextrous use of two decision logics. These studies have shown that the emergence of the synergetic use of causation and effectuation is multifaceted and complex, which would come from firm-related factors and individual-related factors.

### Entrepreneurial Metacognition Theory and Synergy

An entrepreneur is a “motivated tactician” (Fiske and Taylor, 1991, p. 13) who has multiple cognitive strategies (e.g., causal or effectual) available and chooses the most appropriate one through the metacognitive process consciously and prudently (Haynie and Shepherd, 2009).

Even though causation and effectuation follow essentially different principles and assume fundamentally different behaviors, both effectual and causal decision logics are cognitive strategies focused on the “means” of entrepreneurial tasks (Haynie et al., 2010). Here, cognitive strategies refer to organized prior knowledge about individuals and situations aimed at building a meaningful reality and making assessments, judgments, or decisions involving opportunity evaluation, venture creation, or growth (Fiske and Taylor, 1991; Mitchell et al., 2002; Shepherd and Patzelt, 2017). Thus, causal logic is a goal-driven cognitive strategy, and effectual logic is a means-driven cognitive strategy. The choice of causal vs. effectual may depend on the extent to which an entrepreneur employs the metacognitive process (Haynie et al., 2010).

According to the study by Schraw and Dennison (1994, p. 460), metacognition is “the ability to reflect upon, understand, and control one’s learning.” In the context of entrepreneurship, metacognition describes a higher-order cognitive process that “reflects one’s awareness and control over the knowledge structures that are employed to make assessments, judgments, or decisions” (Haynie et al., 2010, p. 220). Metacognitive processes monitor cognitive enterprises that proceed through the actions and interactions among several factors (Flavell, 1979). The four factors are critical building blocks of the metacognitive process, namely, metacognitive knowledge, metacognitive experience, metacognitive choice, and metacognitive monitoring (Haynie et al., 2012).

The idea of metacognition is helpful in our investigation because the metacognition process describes the learning process of an entrepreneur, which can contribute to the cognitive flexibility of an individual. In this research, we followed the conceptualization of Rothman and Melwani (2017) and defined cognitive flexibility as the abilities of entrepreneurs to broaden the scope of their attentional span to attend to divergent perspectives but and engage in a balanced consideration of those perspectives.

Through the active reflections of the awareness of an individual and control over the cognitive strategies employed to make assessments, judgments, or decisions, an entrepreneur can learn and incorporate new information into their consciousness and make better judgments in their selection of cognitive strategy (causal or effectual) (Haynie et al., 2012; Lynch and Corbett, 2021). In brief, decision-makers who engage in metacognitive processes are more likely to recognize multiple alternative cognitive strategies, evaluate those alternatives consciously, and adopt the one to achieve desired outcomes. To summarize, we formally stated as follows:

**Hypothesis 1: Cognitive flexibility of entrepreneurs positively relates to their synergetic use of causal and effectual logic.**

### Emotional Complexity and Synergy

Since the entrepreneurial context is extreme (in terms of time pressure and uncertainty) and an entrepreneur often highly identifies with the venture (Cardon et al., 2005), the entrepreneurial process is filled with substantial emotional experience. In other words, entrepreneurship is an emotional journey (Cardon et al., 2012).

According to “affect as information theory,” emotion is often associated with confidence or doubt about cognitively accessible information, leading to greater or lesser reliance on their current beliefs, expectations, and inclinations of individuals (Clore, 2016; Clore et al., 2018). For example, positive emotions may “promote top-down, theory-based processing in which one relies on cognitively accessible information (e.g., knowledge, beliefs, stereotypes, expectations, primed thoughts)” (Clore et al., 2001). Negative emotions may “promote bottom-up, data-based processing, in which one relies on data from the external environment rather than on internal cognitive constructions” (Clore et al., 2001). In brief, the emotional experience works as an alarm system, which “not only guides judgments and decisions but also guides attention and styles of thinking” (Clore and Bar-Anan, 2007).

However, emotional experience is rarely pure and simple but often mixed and complex (Grossmann and Ellsworth, 2017). People typically report several different or even opposite emotions when describing their feelings (Larsen et al., 2017). According to the emotional complexity theory, feeling a wide range of emotions would provide valuable information about the features of the situation and allow for a more informed prediction of future actions (Berrios, 2019; O’Toole et al., 2020). We followed Barrett et al. (2001) and conceptualized emotional complexity as the ability to have a granular experience of, or variety in, experienced emotions during the emotional episode of an individual.

Individuals differ in their emotional complexity (Kang and Shaver, 2004; Grossmann et al., 2016). Some may experience emotions in a highly differentiated and granular manner, clearly distinguishing between or reporting a great variety of positive or negative discrete emotions; others may experience emotions in a relatively undifferentiated manner, treating a range of like-valence terms as interchange (Lindquist and Barrett, 2008). For example, when entrepreneurs encounter a critical challenge during their project pitch, they may interpret it as evidence of their incompetence and think their project doom and gloom. A simple emotion, such as sorrow, was identified.

In contrast, entrepreneurs with emotional complexity would have mixed emotional experiences. They may first interpret the challenge as a signal of their incompetence and feel a litter sorrow. Then, they reconsider the situation and their previous related emotional knowledge; they categorize the challenge as a test of their pressure-bearing capacity and guess that the funder might be interested in their project. Finally, they felt energized or even pleased to meet the challenge.

Furthermore, according to the entrepreneurial metacognitive theory, emotional experience is an essential type of metacognitive experience (Haynie et al., 2010). It serves as a conduit through which previous emotions may be employed as resources, given the process of making sense of entrepreneurial tasks (Flavell, 1979). Thus, entrepreneurs, who have the granular experience of, or variety in, experienced emotions during the emotional episode, would be more active in their metacognitive processes. They would reckon at the most appropriate cognitive strategy and adapt to the entrepreneurial task more frequently (Rees et al., 2013; Rothman and Melwani, 2017; Grossmann et al., 2019).

In summary, as “the selection of causal versus effectual reasoning may depend, in part, on the extent to which an entrepreneur employs metacognitive processes” (Haynie et al., 2010, p. 225), an entrepreneur with emotional complexity would be more likely on the road to the synergetic use of decision logic through more active metacognitive functioning. Thus, we proposed the following hypothesis:

**Hypothesis 2: Emotional complexity of entrepreneurs positively relates to their synergetic use of causal and effectual logic.**

The human brain works as an active “Bayesian filter” to optimize energy efficiency by anticipating the needs of the body in a situation and preparing emotions and actions to meet those needs in advance (Hoemann et al., 2017). An individual with more granular prior knowledge would have a more accurate posterior prediction of incoming sensory inputs (Grossmann et al., 2016). Similarly, an entrepreneur with a more granular perception of emotions would have more concrete and nuanced information about the situation (Grossmann and Ellsworth, 2017). This information would enable him to expand his cognitive scope, recognize multiple cognitive strategies for his task, evaluate these alternatives consciously, and adopt the most appropriate one flexibly to achieve desired outcomes (Rothman and Melwani, 2017).

Moreover, an emotional-complex entrepreneur is more likely to have extra regulatory resources for the metacognition process. Emotions are related to the most appropriate categories for the sensory experience aroused by external or internal events, which would prepare an existing policy for an individual to take action (Hoemann and Feldman Barrett, 2019). While encountering emotional events, an entrepreneur with emotional complexity would be equipped with a ready-made policy. He would save self-regulation resources for himself and have more opportunities to participate in other regulation processes (such as the metacognitive cognition process) (Kashdan et al., 2014; Erbas et al., 2019).

To summarize, an entrepreneur with emotional complexity would have more accurate and prudent predictions for his sensory experience and more regulatory resources for his metacognitive process. He would view his cognitive strategies from multiple directions and know more about the pros and cons of each decision logic. He would be more agile to switch his cognitive strategy to fit the dynamic changes in the environment. Thus, we formally stated as follows:

**Hypothesis 3: Emotional complexity of entrepreneurs positively relates to their cognitive flexibility.**

**FIGURE 1:**
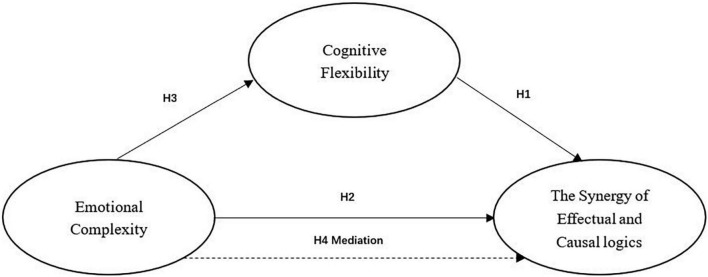
Conceptual model.

Therefore, we proposed a theoretical model in which cognitive flexibility, as a mediator, connects the emotional complexity of entrepreneurs to their collaborative use of the two decision logics (see also Figure 1).

**Hypothesis 4: Cognitive flexibility mediates the positive relationship between emotional complexity and the synergetic use of effectual and causal logic.**

## Methodology

### Data and Sample

We tested our theoretical model using the data collected from entrepreneurs in Sichuan, a southwestern province of the People’s Republic of China (PRC). We recruited entrepreneurs who attended the small business owner training program from a local university and invited them to complete the questionnaire. To ensure the quality of the survey, we told the participants that the purpose of this research was academic and promised that the company and personal information would not be released to third parties for commercial use. Furthermore, we asked the participants to leave their contact information and assured them that we would share the research findings with them in the future if they completed the questionnaire. During one lecture of the training program, 265 entrepreneurs attended, and 236 completed our questionnaires (response rate of 89.05%). After eliminating invalid samples, we retained 218 questionnaires (valid response rate of 82.64%). The average age of participants is 27.84 years (SD = 7.53), and 57.1% of them are female. The majority of our participants are highly educated, 32% with a college diploma and 53.9% with a bachelor’s degree; the average firm age is 2.47 years (SD = 3.35), and the average number of employees is 15.85 (SD = 15.85); the companies primarily engage in culture and art (16%), information technology (12.3%), and wholesale and retailing (12.3%). The demographic features of our participants were similar to those of relevant studies from China (Cai et al., 2017; Yu et al., 2018).

### Measures

The questionnaire used in this research is based on the related theoretical and empirical literature. The questionnaire was administered in Chinese but originally developed in English. We translated the questionnaire from English to Chinese by the translation-back procedure of Brislin (1980) to ensure language equivalence and face validity. Unless otherwise indicated, the scales used in this research are 7-point Likert scales ranging from 1 (strongly disagree) to 7 (strongly agree).

#### Dependent Variable

We measured causation using a scale adapted from Chandler et al. (2011) (6 items, α = 0.910), which is widely accepted and recognized with content validity, face validity, predictive validity, and construct validity (Alsos et al., 2016; Smolka et al., 2018; Yu et al., 2018).

We measured effectuation using a scale adapted from Chandler et al. (2011), which comprises four subdimensions of affordable loss (two items, α = 0.744), flexibility (three items, α = 0.774), experimentation (three items, α = 0.714), and commitments (two items, α = 0.682). Following the practice of Smolka et al. (2018) and Yu et al. (2018), we aggregated the effectuation measure by calculating the average of four subdimensions (α = 0.837).

Following the practice of Braun and Sieger (2021), we used a multiplicative operationalization of causation and effectuation to reflect our argument that the two decision logics are orthogonal and non-substitutable. Specifically, we first calculated the average of causation items and the average of effectuation items and then used the product of two variables to measure the synergetic use of causation and effectuation. This approach has also been used previously by operationalizing organizational ambidexterity to represent the two complementary factors (He and Wong, 2004; Cao et al., 2009; Alsos et al., 2020).

#### Independent Variable

Emotional complexity is conceptualized as the ability of an individual to have a granular experience of, or variety in, experienced emotions during the emotional episode. Accordingly, we adapted the Range and Differentiation of Emotional Experience Scale (RDEES) developed and validated by Kang and Shaver (2004) to measure emotional complexity. An example item of emotional complexity states as follows: “I am good at distinguishing subtle differences in the meaning of closely related emotion words.” We aggregated the emotional complexity measure by combining all items into one construct (six items, α = 0.874).

#### Mediator Variable

Cognitive flexibility is defined as the ability of individuals to broaden the scope of their attentional span to attend to divergent perspectives and engage in a balanced consideration of those different perspectives (Nijstad et al., 2010; Kleiman and Hassin, 2013). Cognitive flexibility was measured by adapting the scale developed and validated by Martin and Rubin (1995), which has been used and validated in many studies (Martin and Anderson, 1998; Martin et al., 2011; Dheer and Lenartowicz, 2019; Kiss et al., 2020). Examples of items in the scale state as follows: “I can find workable solutions to seemingly unsolvable problems,” “I am willing to listen and consider alternatives for handling a problem,” and “I have the self-confidence necessary to try different ways of behaving.” We aggregated the cognitive flexibility measure by combining all items into one construct (five items, α = 0.833).

#### Control Variables

We controlled for several background characteristics of the entrepreneur that would influence the choice of decision logic. At the individual level, we controlled for the gender of an entrepreneur (0 = male, 1 = female), age (in years), and education (1 = middle school or below, 2 = college, 3 = bachelor’s degree, 4 = master’s degree, and 5 = Ph.D.). We also controlled for the prior work experience of an entrepreneur (0 = new to the industry and 1 = have worked in the industry).

Firm age (in years), size (number of full-time employees), industry sector, and subjective performance are control variables at the firm level. Prior research has found that the industry sector (Reymen et al., 2015; Yu et al., 2018) and venture performance (Smolka et al., 2018) are related to the synergetic use of decision logic. Accordingly, the following eight broad industry sectors are used as control variables: agriculture, information technology, wholesale and retailing, special technical service, healthcare, culture and art, construction and manufacturing, and a combined sector for the rest. We also included the subjective assessment of venture performance of entrepreneurs in comparison with their peers.

### Common Method Variance

Following the suggestion by Podsakoff et al. (2003) on common method bias, we employed procedural approaches to minimize the potential impact of common method bias at the design stage and conducted additional statistical tests at the data analysis stage.

The established instruments were used to measure our constructs at the design stage, and the scales were arranged in counterbalanced question order. At the data analysis stage, Harman’s one-factor and latent-factor tests were used to evaluate the effect of common method variance.

Harman’s one-factor test showed that the first factor explained 38.68% of the variance. A confirmatory factor analysis (CFA) with the independent, dependent, and mediating variables revealed a good fit [χ^2^(303) = 538.721; CFI = 0.922; RMSEA = 0.060], and the CFA result for one factor on which all items loaded showed a significantly worse fit [χ^2^(350) = 1,269.336; CFI = 0.706; RMSEA = 0.110; difference in χ^2^ = 730.615; df = 47, *p* < 0.001].

These results show that the common method bias may not be a serious problem in this research.

## Results

Table 1 reports the mean, SD, and Pearson correlation of all research variables. Consistent with our theoretical proposition, emotional complexity is significantly associated with cognitive flexibility and the synergetic effect of the two decision logics. As in other related studies (Smolka et al., 2018; Yu et al., 2018; Braun and Sieger, 2021), the correlations between causation and aggregated effectuation or its subdimensions are relatively high.

**TABLE 1:** Descriptive statistics and Pearson correlations (*N* = 218).

		Mean	SD	1	2	3	4	5	6	7	8	9	10	11	12	13	14	15	16
1	Emotional complexity	4.99	1.06	(0.874)															
2	Cognitive flexibility	5.01	0.93	0.525**	(0.833)														
3	Causation	5.05	1.06	0.506**	0.668**	(0.910)													
4	Affordable loss	5.24	1.21	0.402**	0.379**	0.420**	(0.744)												
5	Flexibility	4.92	1.09	0.388**	0.560**	0.626**	0.442**	(0.774)											
6	Experimentation	4.39	1.27	0.329**	0.461**	0.574**	0.173*	0.443**	(0.714)										
7	Precommitments	5.01	1.12	0.334**	0.463**	0.497**	0.330**	0.550**	0.411**	(0.682)									
8	Aggr. effectuation	4.85	0.87	0.487**	0.636**	0.730**	0.604**	0.834**	0.757**	0.734**	(0.837)								
9	Causation × Aggr. effectuation	25.14	8.89	0.540**	0.704**	0.927**	0.538**	0.782**	0.715**	0.647**	0.921**	1.000							
10	Gender	0.57	0.50	0.029	–0.080	–0.094	0.140*	–0.080	−0.201**	–0.037	–0.089	–0.111	1.000						
11	Age	27.84	7.53	0.039	–0.007	–0.057	0.186**	0.009	−0.197**	–0.001	–0.032	–0.063	0.494**	1.000					
12	Education	2.66	0.71	0.112	0.142*	0.094	–0.034	–0.012	–0.062	–0.027	–0.048	0.037	–0.054	–0.034	1.000				
13	Prior experience	0.48	0.50	–0.099	–0.084	–0.066	–0.066	–0.036	–0.032	–0.100	–0.071	–0.073	0.065	0.086	–0.099	1.000			
14	Firm age	2.47	3.35	0.008	0.031	0.026	0.122	0.063	–0.023	0.031	0.055	0.034	0.369**	0.629**	–0.131	0.147*	1.000		
15	Firm size	15.85	18.19	0.077	0.045	0.062	0.131	–0.014	–0.013	–0.040	0.015	0.033	0.405**	0.556**	–0.074	0.096	0.579**	1.000	
16	Performance	4.27	1.26	–0.026	0.142	0.137	0.164*	0.189*	0.024	0.179*	0.171*	0.158*	0.143	0.297**	0.000	–0.082	0.266**	0.295**	1.000

**Correlation is significant at the 0.01 level (2-tailed). *Correlation is significant at the 0.05 level (2-tailed). Reliability of the measure in parentheses (Cronbach’s alpha). Aggr = aggregated.

Moreover, the results also show that the age of the founder is positively associated with the affordable loss subdimension of effectuation and negatively associated with experimentation. The education of the founder is positively associated with cognitive flexibility and negatively associated with the experimentation subdimension of effectuation.

Hierarchical linear regression analysis was used to test our hypotheses, and the bootstrap method was used to test the mediating effect of the theoretical model. The results of the regression and mediation analyses are presented in Tables 2, 3.

**TABLE 2:** Results of regression analysis.

	Model 1			Model 2			Model 3			Model 4			Model 5		
	Cognitive flexibility			Cognitive flexibility			Synergetic use			Synergetic use			Synergetic use		
	*Coeff*	*SE*	*p*	*Coeff*	*SE*	*p*	*Coeff*	*SE*	*p*	*Coeff*	*SE*	*p*	*Coeff*	*SE*	*p*
Intercept	4.609	0.432	0.000	2.292	0.448	0.000	25.227	4.221	0.000	2.625	4.373	0.549	–10.845	3.757	0.004
***Control variables***															
Gender	–0.295	0.164	0.074	–0.283	0.137	0.041	–3.216	1.604	–2.004	–3.092	1.342	0.022	–1.431	1.088	0.190
Age	–0.013	0.013	0.301	–0.010	0.011	0.341	–0.190	0.126	–1.516	–0.160	0.105	0.129	–0.100	0.084	0.238
Education	0.168	0.093	0.073	0.085	0.078	0.280	0.419	0.907	0.462	–0.389	0.764	0.611	–0.887	0.614	0.150
Firm age	0.013	0.026	0.629	0.015	0.022	0.510	0.233	0.259	0.900	0.250	0.216	0.249	0.164	0.174	0.345
Firm size	0.003	0.005	0.479	0.000	0.004	0.919	0.032	0.044	0.720	–0.003	0.037	0.930	–0.001	0.030	0.974
Prior experience	–0.060	0.139	0.669	0.069	0.117	0.555	–0.694	1.359	–0.511	0.563	1.146	0.624	0.155	0.919	0.866
Performance	0.120	0.058	0.040	0.143	0.048	0.004	1.334	0.565	2.362	1.559	0.473	0.001	0.719	0.388	0.066
***Independent variable***															
Emotional complexity				0.465	0.053	0.000				4.536	0.518	0.000	1.802	0.498	0.000
***Mediator variable***															
Cognitive flexibility													5.878	0.592	0.000
*R* ^2^		0.077			0.359			0.082			0.362			0.593	
*Adjusted R^2^*		0.040			0.330			0.045			0.332			0.571	
*F*		2.094			12.245			2.243			12.389			28.117	

*N = 184. Since the regression results do not depend on the industry dummy variables, we reported the model without the industry variables for simplicity. Unstandardized regression coefficients are reported.*

**TABLE 3:** Results of mediation analysis.

Effect	Estimate	SE	95% CI Lower	95% CI Upper
Indirect Effect (Emotional Complexity → Cognitive Flexibility → Synergetic Use of Causation and Effectuation)	2.734	0.449	1.912	3.681
Direct Effect (Emotional Complexity → Synergetic Use of Causation and Effectuation)	1.802	0.498	0.819	2.785

*The effects are estimated by bootstrapping with 5,000 iterations.*

We followed the procedures developed by Preacher and Hayes (2008) to test the mediating effect in our model. First, we regressed cognitive flexibility on the control variables as our base model and then added emotional complexity (Model 2) to test Hypothesis 2. Then, we regressed the synergetic use of causation and effectuation on the control variables, cognitive flexibility, and emotional complexity in sequence (from Models 3 to 5) to test Hypotheses 1 and 3. Finally, we used the “PROCESS” macro suggested by Hayes (2018) to assess the indirect effects of emotional complexity on the synergetic use of two decision logics (Hypothesis 4).

Table 2 shows that entrepreneurs with higher emotional complexity are positively associated with higher cognitive flexibility (*b* = 0.465, *p* < 0.000); thus, Hypothesis 3 is supported. In addition, after controlling the influence of cognitive flexibility of entrepreneurs (*b* = 5.878, *p* = 0.000), there is still a positive association between emotional complexity and the synergy of causation and effectuation (*b* = 1.802, *p* < 0.000); thus, Hypotheses 1 and 2 are supported.

In Table 3, the 95% CIs for the indirect effect of emotional complexity obtained by the bootstrapping with 5,000 iterations do not include zero (*b* = 2.7343, SE = 0.449, and CI = 1.9123, 3.6809), which means that the mediating effect of cognitive flexibility can be assumed. Thus, Hypothesis 4 was supported.

## Discussion and Conclusion

Recently, several empirical studies have shown the synergetic effect of causal and effectual logic on venture performance (Smolka et al., 2018; Braun and Sieger, 2021; Galkina et al., 2021). However, we still know less about how we can get it. To fill this gap, we introduced the entrepreneurial metacognition and emotional complexity theory and established a model for the emergence of synergetic use of causal and effectual logic. We found that an entrepreneur with emotional complexity is more likely to use decision logic in tandem, and cognitive flexibility partially mediates this relationship.

### Theoretical Contributions

Our research makes several theoretical contributions to the literature.

First, our core contribution is the introduction of emotional complexity as a critical antecedent to the synergetic use of causation and effectuation. Responding to the call from Shepherd (2015) to link emotions with entrepreneurial decision-making, this study establishes the theoretical and empirical links between emotional complexity and decision logic. It adds a new perspective to the burgeoning literature on effectuation (Matalamäki, 2017). More specifically, an entrepreneur with emotional complexity interprets his sensory experiences more accurately and has more regulatory resources for metacognitive functioning. They are more likely to use two entrepreneurial logics in tandem to tackle entrepreneurial tasks. These findings confirm that synergy is driven not only by firm-related factors but also by individual-related emotional factors (Braun and Sieger, 2021).

Second, by introducing emotional complexity theory, we addressed the call to shift from single emotion to complex emotions (Gielnik et al., 2021) and extended the scope of emotion research in entrepreneurship research. Although scholars are aware that emotions play an essential role in the cognition of an entrepreneur, few researchers have noted that the emotional experience of an entrepreneur is seldom simple but often complex (Grossmann et al., 2016). During the venture creation process, the emotional experience of an entrepreneur is like a rollercoaster (De Cock et al., 2020). Moreover, entrepreneurs differ in their emotional complexity (Kang and Shaver, 2004). Thus, we argued that complex emotions, but not simple emotions, impact the choice of cognitive strategy of an entrepreneur. Complex emotion alerts entrepreneurs to the contradictory and conflicting elements in the environment and contributes to their choice of the most appropriate cognitive strategy.

Finally, we advanced the entrepreneurial metacognitive theory by developing a novel theoretical link between emotional complexity, cognitive flexibility, and decision logic. Previous studies view the synergy of casual and effectual as an outcome of metacognitive functioning and propose that metacognitive experiences are especially likely to occur during intense emotional experiences (Haynie and Shepherd, 2009; Haynie et al., 2010, 2012). This study extends this strand of literature by addressing the complexity of emotional experience as an antecedent to cognitive flexibility, enabling us to have a more nuanced view of the role of emotion in the metacognitive process of an entrepreneur. Specifically, an entrepreneur with granular emotional knowledge has a more accurate and complex emotional experience, which stimulates their metacognitive awareness, and results in them being more active in the choice of the most appropriate cognitive strategy for entrepreneurial tasks.

### Managerial Implications

The results of this study should also raise entrepreneurs and educators’ awareness of the benefits of granular emotional experiences (Barrett, 2017a). We hope that our research findings will induce entrepreneurs to be more sensitive to their emotional experiences and interpret emotional information accurately and adaptively. We also expect educators to incorporate the concept of emotional complexity into their curriculum for entrepreneurs and innovators (Honig, 2004; Wu and Chen, 2021).

### Limitations and Future Directions

There are some limitations to this study that might open future research avenues.

First, since the research design of this study is based on a cross-sectional survey, we can only confirm the correlation but not causality. Studies using longitudinal or experimental designs are needed to replicate our findings. Specifically, researchers could use the mediating experimental design (Stone-Romero and Rosopa, 2010; Pirlott and MacKinnon, 2016) to test the mediating effect of cognitive flexibility between emotional complexity and synergetic use of decision logic.

Second, we measured emotional complexity by asking entrepreneurs to characterize their emotional experiences in global and retrospective terms. There are several measurements for assessing the ability of an individual to identify or recognize affective feelings as differentiated emotional experiences (O’Toole et al., 2020). We recommend that future studies use other measurements to verify our model. Specifically, experience sampling and think-aloud methods can be adopted to observe how entrepreneurs report their emotional experiences on a moment-to-moment basis (Davison et al., 1997; Hoemann et al., 2021).

Third, we conceptualized synergy as the conscious and proactive choice of decision logic of an entrepreneur. However, synergy might occur passively. For example, an entrepreneur may have to adopt casual and effectual logic in tandem to meet venture capital or incubator requirements (Frese et al., 2020). Further research could develop a model to integrate the proactive and passive adoption of two decision logics.

Fourth, this research provides the emotional complexity of an entrepreneur to explain the emergence of synergy in the mind of an individual. However, synergy may arise from integrating team members with differentiated decision logic (Alsos et al., 2020). Therefore, future research could extend the model from the individual level to the team level.

## Data Availability Statement

The raw data supporting the conclusions of this article will be made available by the authors, without undue reservation.

## Ethics Statement

The studies involving human participants were reviewed and approved by the Southwestern University of Finance and Economics Ethics Committee. The participants provided their written informed consent to participate in this study.

## Author Contributions

HC: literature review, methodology, and revision. QX: conceptualization, data collection, manuscript writing, and project administration. Both authors contributed to this study and approved the submitted version.

## Conflict of Interest

The authors declare that the research was conducted in the absence of any commercial or financial relationships that could be construed as a potential conflict of interest.

## Publisher’s Note

All claims expressed in this article are solely those of the authors and do not necessarily represent those of their affiliated organizations, or those of the publisher, the editors and the reviewers. Any product that may be evaluated in this article, or claim that may be made by its manufacturer, is not guaranteed or endorsed by the publisher.

## Footnotes

1 Sarasvathy (2001) proposed the following three types of means available to the entrepreneurs for their entrepreneurial task: who they are, what they know, and whom they know.

2 Cognitive strategy refers to the “knowledge structures that people use to make assessments, judgments, or decisions involving opportunity evaluation, venture creation, and growth” (Mitchell et al., 2002, p. 97).
